# Histopathology pattern and survival analysis of patients with kidney biopsy in the top end of Northern Australia from 2007 to 2020

**DOI:** 10.1186/s12882-022-03011-2

**Published:** 2022-12-01

**Authors:** Kim Ling Goh, Asanga Abeyaratne, Shahid Ullah, Chris Rissel, Kelum Priyadarshana

**Affiliations:** 1 Department of Nephrology, Royal Darwin Hospital, Darwin, NT Australia; 2 College of Medicine and Public Health, Flinders University, Darwin, NT Australia

**Keywords:** Kidney biopsy, Northern territory, Indigenous, Survival analysis, Histopathology

## Abstract

**Aim:**

Royal Darwin Hospital (RDH) is the main tertiary hospital that has performed more than 600 biopsies since its establishment. Although Indigenous people in Australia’s Northern Territory (NT) has the highest rate of renal replacement therapy, the histopathology pattern of their renal diseases is still under discussed. We aimed to analyse the histopathology pattern of patients undergoing renal biopsy in RDH from June 2007 to June 2020. Secondary aims include clinical indication and survival analysis of patients with kidney biopsies.

**Methods:**

We conducted a retrospective cohort study on all native kidney biopsy reports from patients over the age of 16, from June 2007 to June 2020. Descriptive statistics was used to summarise age, sex, indigeneity, histopathological pattern, and mortality. Categorical values were expressed as absolute frequencies and percentages. Survival analysis was performed using multivariate analyses and Cox proportional hazard regression model.

**Results:**

There were 364 native renal biopsies included in the analysis. Sub-nephrotic proteinuria was the most common clinical indication for kidney biopsy (*n* = 160,47.8%). Diabetes nephropathy (DN) was the most common pathological finding (*n* = 71,12.8%). Indigenous population who had dialysis performs poorly compared to their non-indigenous counterpart (HR 2.37,95% CI 1.53–3.67,*p* < 0.001).

**Conclusion:**

Diabetic nephropathy is the most common native kidney biopsy in the NT with higher mortality among indigenous patients. This study supports the previous findings of indigenous female excess, younger age of kidney disease requiring kidney biopsy, and excess of diabetic nephropathy in the top-end of the NT. It can be speculated that some diabetic patients had atypical features prompting a biopsy.

## Introduction

Since the early 1950s, renal biopsy has become the gold standard for the diagnosis of pathologic renal diseases [1]. According to ANZDATA 2019, Northern Territory (NT) has the highest prevalence of end stage renal failure (ESRF) in the Australia [2]. However, there is no study that has provided the overall analysis of renal pathology detected in renal biopsies in adult patients in the NT. Kidney biopsy data is even more limited among Indigenous population. Issues like cultural miscommunications, logistic problems can have potentially detrimental effect on the delivery of quality healthcare services for Indigenous population [2]. Distribution and incidence of kidney diseases vary globally over time and may be highly influenced by local standard practice patterns. Hence, it is crucial to have a population-based effort to inform trends in disease spectrum. These data could be informative in making health policies and resource allocation, facilitate funding in research and refine pre-test probabilities in clinical practice [3].

Our primary aim of the study is to assess the pattern of renal pathology detected in renal biopsies of adult patients in the NT from 2012 to 2020.

Our secondary aim of the study is to evaluate the clinical indications of kidney biopsy and survival analysis of the main histology group (diabetic nephropathy, focal segmental glomerulosclerosis, IgA nephropathy and hypertensive renal diseases). It will help clinicians to stratify patient population in terms of necessity and yield of renal biopsy. This data will particularly helpful with interpretation of indigeneity and renal biopsy result.

## Methods

This retrospective population-based cohort study was approved by Human Research Ethics Committees (HREC) of the Menzies School of Research and the Top End Health Service Research Governance Office, Northern Territory, Australia (HREC 20,203,831).

We collected data from the Electronic Medical Record (EMR) of the Top End Health Service (TEHS).

All samples were assessed by light microscopy, immunofluorescence and electron microscopy. Every renal biopsy was reported by a renal pathologist at the Queen Elizabeth Hospital in South Australia. Unfortunately, we could not retrieve the specific pathologist who are in charge of all the kidney biopsies.

Other information we extracted included age of patient, ethnicity (indigeneity), date of procedure, clinical indication and histopathological diagnosis, date of dialysis or death from the electronic medical record. Inclusion criteria were all adults (aged 18 years old and above), who had kidney biopsy in TEHS both outpatient or inpatient setting. The exclusion criteria were renal biopsies done on transplanted kidney, suboptimal sample or inconsistent demographics. Suboptimal sample is defined by a kidney biopsy sample obtained is not adequate for the pathologist to make any make diagnosis. In case of an unclear histopathological diagnoses, the images were interpreted independently by two experienced nephrologists. Any difference in opinion was sorted with discussion and consensus.

The renal pathology derived from renal biopsies was individually reviewed and categorised by the principal investigator according to the World Health Organisation recommendations. The main category of histopathology pattern of renal biopsy includes diabetic mellitus nephropathy (DMN), Focal Segmental Glomerulosclerosis (FSGS), IgA Nephropathy (IgAN), glomerulomegaly, Hypertensive Renal Disease (HRD), Proliferative Glomerulonephritis, Post Infective Glomerulonephritis (PIGN), Membranoproliferative Glomerulonephritis (MPGN), Acute Tubular Necrosis (ATN), Acute Interstitial Nephritis (AIN), Minimal Change Disease (MCD), ANCA associated vasculitis (AAV), Thin Basement Membrane Nephropathy (TBMN), AA amyloid, AL Amyloid, Thrombotic Microangiopathy (TMA), myeloma kidney, anti-Glomerular Basement Membrane (GBM), Scleroderma Renal Crisis (SRC), Contrast-induced Nephropathy (CIN), C3-Glomerunephritis (C3GN), Interstitial fibrosis and tubular atrophy (IFTA), Lupus Nephritis (LN), Alport Syndrome (AS) and Renal Ischemia (RI). The renal pathology was interpreted in the context of clinical features as written on the pathology form. Biopsy results with more than one histopathology diagnosis were assigned to the predominant group.

The clinical indications of kidney biopsies were interpreted and categorised into six different categories as defined in Table 1. The category of clinical indication includes sub-nephrotic range proteinuria, nephrotic syndrome, nephritic syndrome, renal impairment of unknown cause, isolated painless haematuria and acute kidney injury. Patients presenting with more than one clinical feature were assigned to all corresponding groups.

**Table 1:** Clinical indication and definition [4]

Clinical category	Definition
Nephrotic syndrome	A urine protein excretion of more than 3.5 g per 24 h or a random urine protein-to-creatinine ratio more than 3000 mg/g in an adult
Sub-nephrotic proteinuria	Albumin-Creatinine Ratio of < 250 mg/mmol or Protein-Creatinine Ratio < 300 mg/mmol
Nephritic syndrome	A constellation of haematuria, proteinuria, hypertension and/or acute kidney injury (AKI)
acute kidney injury (AKI)	Increase in serum creatinine of ≥ 26.5 μmol/L within 48 h and labelled as AKI or rapid increase in creatinine in the clinical scenario
Renal Impairment of unknown cause	Any new presentation of chronic kidney diseases (defined by glomerular filtration rate less than 60 ml/min/1.73m2), without an obvious cause (determined by treating nephrologist)
Isolated painless haematuria	Presence of painless haematuria in patients

In terms of survival analysis, we only considered patients with the main histology groups including DMN, FSGS, IgA nephropathy and HRD. The renal endpoint analysed in this study included initiation of dialysis, death or end of study. In patients who experienced both dialysis and death, the earliest of two was taken as censored date. The follow up time of this study was 14 years.

A descriptive statistic was used to summarise age, sex, indigeneity and renal pathology. Data were presented as mean +—standard deviation for categorical data or median and interquartile ranges for continuous variables as appropriate. Categorical values were expressed as absolute frequencies and percentages. The age group was stratified into 10–19 years old, 20–29 years old, 30–39 years old, 40–49 years old, 50–59 years old, 60–69 years old, 70–79 years old and 80 years old or above. The data were expressed in pie charts and bar charts. Median survival rates were estimated using the Kaplan–Meier method according to the main histology diagnosis and indigeneity. Hazard ratio were estimated using Cox regression model. All factors associated with outcomes in the univariate analysis were fed into multivariate models, adjusting for age and indigeneity. All statistical tests were two-sided and the threshold for statistical was *p* < 0.05. Statistical analyses were performed using Microsoft Excel and R Project for Statistical Computing 4.2.0.

## Results

### Demographics

**Fig. 1:**
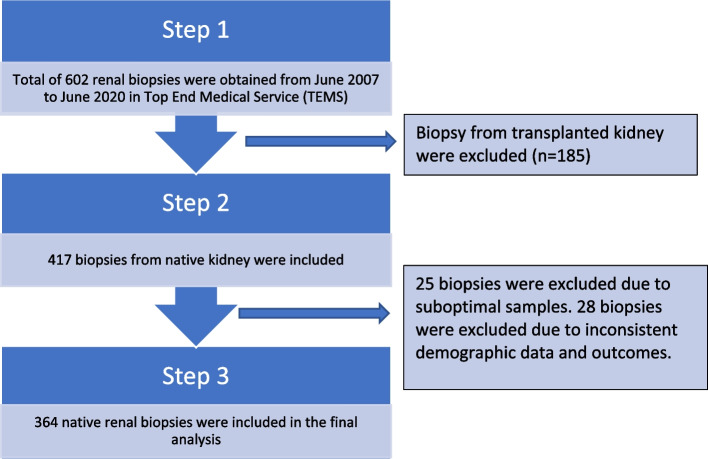
Flow chart of patient selection

In. brief, a total of 602 renal biopsies performed between June 2007 and June 2020 was collected. 185 transplanted renal biopsies were excluded in the early stage of analysis. 25 biopsies were excluded from analysis due to suboptimal histology sample. There were 364 native renal biopsies that we included in the analysis as shown in Fig. 1.

**Fig. 2:**
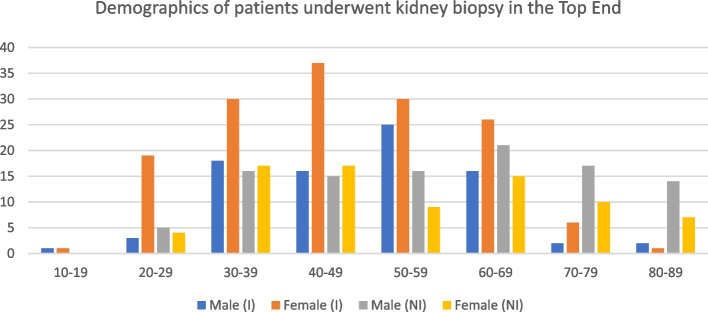
Patient demographics of renal biopsies according to sex and indigeneity. *Footmark: I: indigenous; NI: non-indigenous

Figure 2 shows the patient demographics of renal biopsies according to sex and indigeneity. The age at the time of kidney biopsy ranged from 17 years old to 89 years old, with male (210 cases, 39.6%%) to female (320 cases, 60.4%) of the full cohort, with mean age at the time of biopsy was 49.2 ± 15.5 years for female and 52.7 ± 15.3 years. The mean difference between age between male and female was 3.5 years old (*p* = 0.024). Indigenous female had the greatest number of renal biopsies (*n* = 231 43.6%), compared to the three other main groups. Most kidney biopsies were done in the age group 40–49 years old (18.8% indigenous male, 43.5% indigenous females, 17.6% non-indigenous males and 20.0% non-indigenous females). Most indigenous males who underwent renal biopsies were from the age group of 50 to 59 years old (*n* = 25) while most indigenous females were from the age group of 40–49 years old (*n* = 37). Most non-indigenous males had renal biopsy at the age of 60–69 years old (*n* = 21) while most non-indigenous females had renal biopsy at the age of 30–49 years old (*n* = 34).

### Clinical indications

**Fig. 3:**
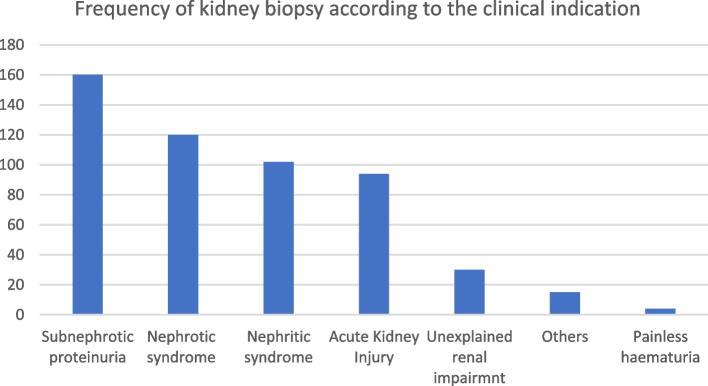
Frequency of renal biopsy according to the clinical indication

Figure 3 shows the clinical indications of renal biopsy. Sub-nephrotic proteinuria was the most common clinical indication for kidney biopsy, accounting of 47.8% (*n* = 160) of all cases, followed by nephrotic syndrome (*n* = 120, 35.8%), nephritic syndrome (*n* = 102, 30.4%), acute kidney injury (*n* = 94, 28.1%), unexplained renal impairment (*n* = 30, 9.0%) and others (*n* = 15, 4.5%). The ‘others’ category included any kidney biopsy in which the treating nephrologist did not specify the indication of the procedure on pathology request form.

### Pathological diagnosis

Diabetes nephropathy (DN) was the most common pathological finding, accounting for 12.8% (*n* = 71), followed by focal segmental glomerulosclerosis (FSGS) (*n* = 47, 8.5%), glomerulomegaly (*n* = 44, 7.9%), IgA nephropathy (*n* = 44, 7.9%) and hypertensive renal disease (*n* = 14, 2.5%). Post-infective glomerulonephritis (PIGN) contributed to 4.5% (*n* = 25) of the renal biopsy in the Northern Territory.

Based on the International Society of Nephrology/Renal Pathology Society classification, the cohort of lupus nephritis (LN) was categorised as follows: class I (*n* = 22, 4.0%), class II (*n* = 8, 1.4%), class III (*n* = 11, 2.0%), class IV (*n* = 17, 3.1%) and class V (*n* = 12, 2.1%).

Diabetic nephropathy was the most common diagnosis among indigenous males (*n* = 20, 2.17%) and indigenous females (*n* = 39, 54.93%). Most non-indigenous males (*n* = 17, 38.64%) have IgA nephropathy while most non-indigenous females (*n* = 13, 27.66%) had focal segmental glomerulosclerosis (FSGS).

In FSGS, indigenous females contributed to the highest count (*n* = 15, 31.91%), followed by non-indigenous females (*n* = 13, 27.66%), indigenous males (*n* = 11, 23.40%) and non-indigenous males (*n* = 8, 17.02%).

Among 25 people with PIGN, 60% (*n* = 15) were from indigenous population, which consists of 10 indigenous males and 5 indigenous females. Males (*n* = 15) had a higher incidence of PIGN than females (*n* = 10).

The majority of IgA nephropathy was found in non-indigenous males (*n* = 17, 38.64%). Indigenous females and non-indigenous females contributed to the same number (*n* = 10, 22.73%). Only 7 indigenous males (*n* = 7, 15.91%) were diagnosed with IgA nephropathy.

Among the patients with normal renal biopsy, the number from each patient group was similar. 4 indigenous males and 3 indigenous females had normal biopsies while 3 non-indigenous males and 4 non-indigenous females had normal renal biopsies in RDH.

**Fig. 4:**
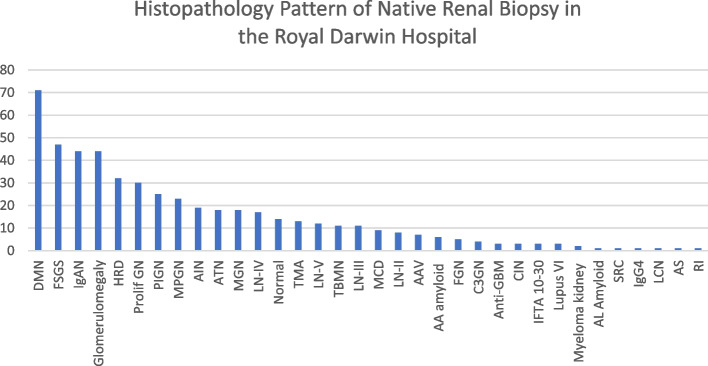
Histopathology pattern of renal biopsy. Footmark: IgAN: IgA nephropathy; ATN: acute tubular necrosis; AIN: acute interstitial necrosis; MCD: minimal change disease; FSGS: focal segmental glomerulosclerosis; MGN: membranous glomerulonephritis; AAV: ANCA-associated vasculitis; PIGN: post-infective glomerulonephritis; Prolif GN: proliferative glomerulonephritis; MPGN: membranous-proliferative glomerulonephritis; DMN: diabetic nephropathy; TBMN: thin-basement membrane nephropathy; TMA: thrombotic microangiopathy; SRC: Scleroderma renal crisis; CIN: Contrast-induced nephropathy; C3GN: C3 glomerulonephropathy; IFTA 10–30: Interstitial fibrosis and tubular atrophy; LN: lupus nephropathy; FGN: Fibrillary glomerulonephritis; LCN: Liver cirrhosis-related nephropathy; AS: Alport Syndrome; HRD: Hypertensive renal disease; RI: Renal ischemia

No indigenous population were diagnosed with AL amyloid, anti GBM, scleroderma, light chain nephropathy and Alport Syndrome. The complete histopathology pattern of native renal biopsy conducted in the Top End is demonstrated in Fig. 4.

### Survival

**Fig. 5:**
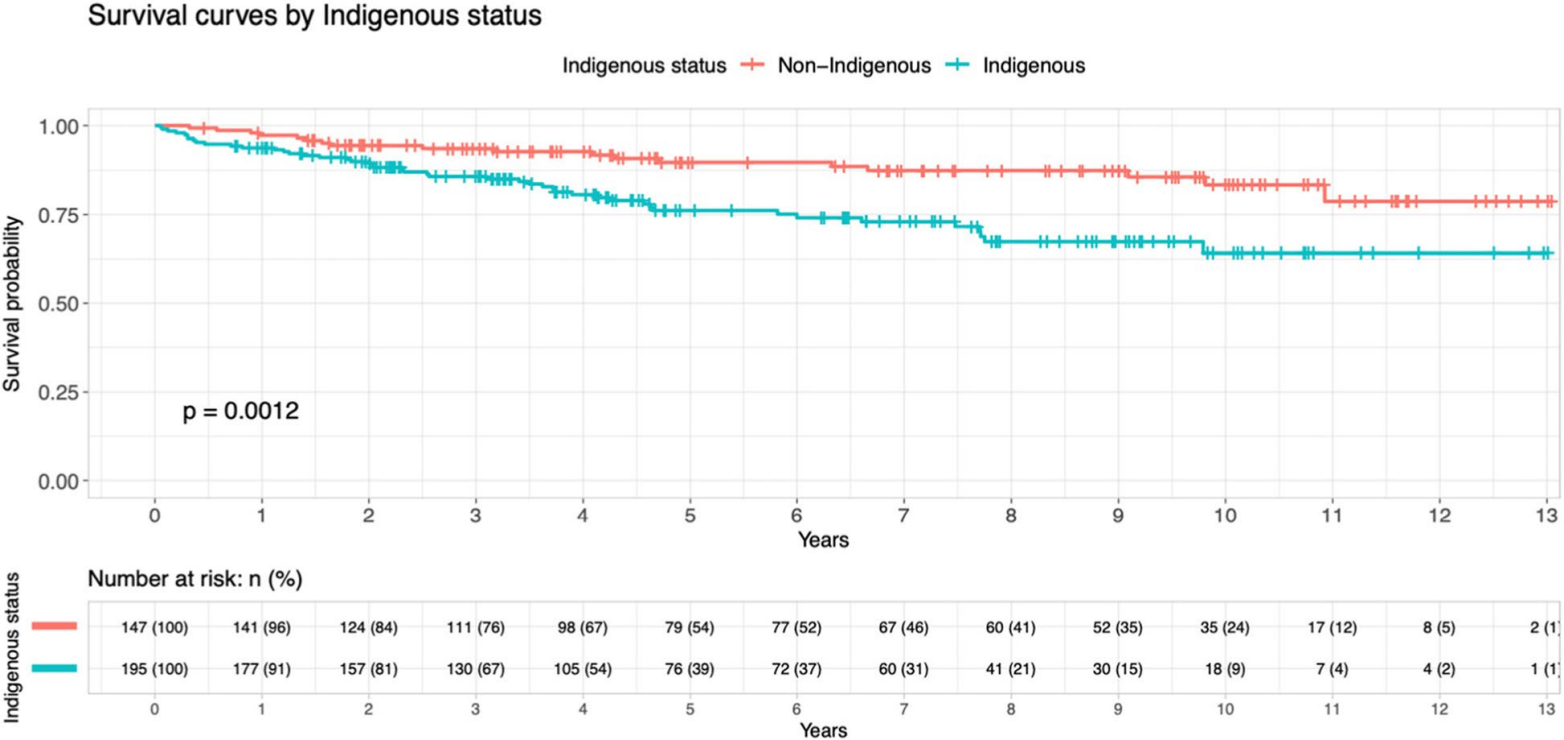
Kaplan–Meier survival curves of patients underwent kidney biopsies by Indigenous status

In total there were 64 deaths (18.2%) and 101 patients who undergone at least 1 dialysis episode (28.8%). Overall, Indigenous population had a significantly poorer survival than non-indigenous population based on Fig. 5. The univariate hazard ratio of indigenous status was 2.41 (95% CI: 1.39–4.18, *p* = 0.002). The hazard ratio from multivariate Cox proportional hazard model survival adjusting for indigeneity, age and sex are shown in Table 2. There was a very strong evidence to show that indigeneity status and high age group were significant factor that led to poorer outcome. Male had slightly worse outcome than female (HR 0.60, 95% CI 0.36–0.98, *p* = 0.043).

**Table 2:** Multivariate Cox proportional hazard model survival, adjusting for indigenous status, sex and age

Characteristics	Hazard ratio	95% confidence interval	*p* value
Indigenous	4.03	2.25–7.22	< 0.001
Age	1.05	1.03–1.07	< 0.001
Sex	0.60	0.36–0.98	0.043

**Fig. 6:**
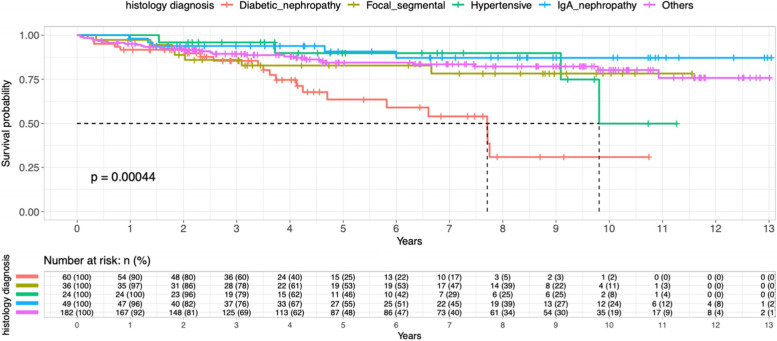
Kaplan–Meier survival curve by histology of kidney biopsy

Figure 6 shows Kaplan–Meier survival curve by histology of kidney biopsy. For practical purpose, we were focusing on the main histology including diabetic nephropathy, focal segmental glomerulosclerosis, hypertensive renal disease and IgA nephropathy. Other histology diagnosis was categorized as ‘others’. Diabetic nephropathy was the reference group and the other groups were compared accordingly. The risk of death was significantly lower than the Diabetic Nephropathy group. Patients with FSGS had 59% lower chance of death (HR 0.41, 95% CI 0.17–0.97, *p* = 0.0043) while patients with hypertensive renal disease had 64% lower chance of death (HR = 0.36, 95% CI 0.12–1,06, *p* = 0.0063).

**Fig. 7:**
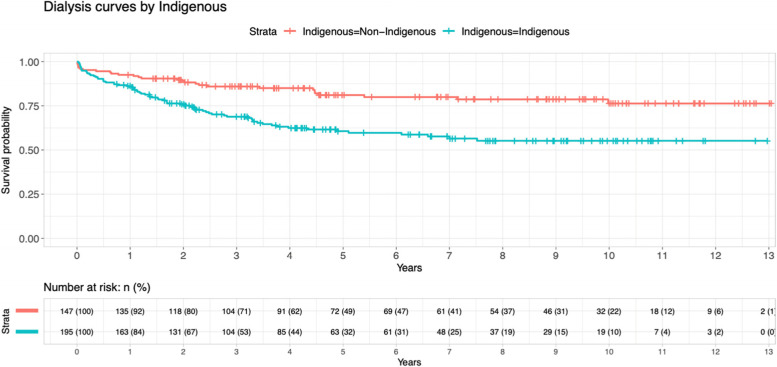
Kaplan–Meier curve for patients undergone dialysis, by indigenous status

Figure 7 and Table 3 shows the survival analysis for patients undergone dialysis, sorted by indigenous status. There was a very strong statistical significance that indigenous population who had dialysis performs poorly compared to their non-indigenous counterpart (HR 2.37, 95% CI 1.53–3.67, *p* < 0.001).

**Table 3:** Shows multivariate cox proportional hazard model survival of patients with main histology diagnosis (Reference group: Diabetic Nephropathy)

Characteristics	Hazard ratio	95% CI	*P* value
Focal segmental glomerulosclerosis	0.41	0.17–0.97	0.0043
Hypertensive renal disease	0.36	0.12–1.06	0.0063
IgA nephropathy	0.20	0.07–0.54	0.001
Others	0.33	0.18–0.59	< 0.001

**Fig. 8:**
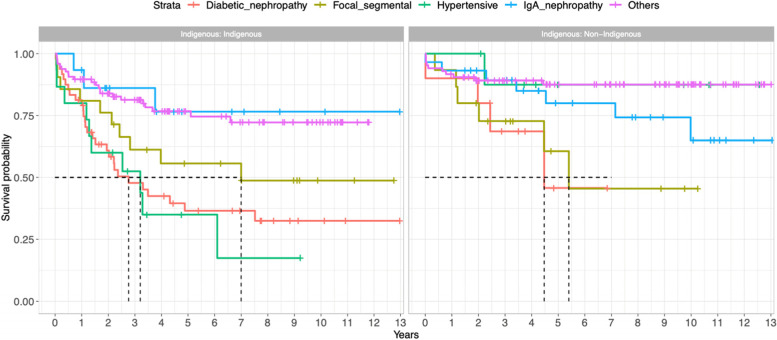
Shows Kaplan–Meier curve for patients who had dialysis, stratified by their indigenous status and histology diagnosis

As shown in Fig. 8 and Table 4 shows the survival analysis for population who had dialysis, stratified by their indigenous status and histology diagnosis. The difference in the survival of dialysed patients was not statistically significant for FSGS, HRD and IgA nephropathy.

**Table 4:** Shows survival analysis for population who had dialysis, stratified by their indigenous status and histology diagnosis (Diabetic nephropathy as the reference group)

Characteristics	Non-indigenous			Indigenous		
	**HR**	**95% CI**	**P value**	**HR**	**95% CI**	***P* value**
**Focal segmental glomerulosclerosis**	0.97	0.27–3.46	> 0.900	0.72	0.33–1.57	0.4
**Hypertensive renal disease**	0.20	0.02–1.79	0.150	1.32	0.63–2.75	0.5
**IgA nephropathy**	0.45	0.12–1.61	0.200	0.31	0.09–1.04	0.058
**Others**	0.23	0.07–0.74	0.014	0.36	0.20–0.66	< 0.001

## Discussion

Currently there are no contemporary large-scale renal biopsy studies addressing the histopathology pattern of kidney diseases in the Northern Territory of Australia. NT is one of the states with the highest prevalence of renal disease in Australia. We retrospectively analysed 364 patients in the NT who underwent native renal biopsy in Royal Darwin Hospital over the past 14 years to explore the epidemiology of kidney disease. This study provides a picture of the current histopathology pattern in this region, which can serve as a useful resource in making hypothesis-driven research and health policy decisions.

The most common age group in our study who had biopsy is 40–49 years old, as contrast to a few studies in Bangladesh [5–7]. In the study by Narsimhan et al., only 10% patients were more than 55 years old [8]. As compared to high income countries, 33% of the patients who had renal biopsy was more than 50 years old. Longer life span and better access to healthcare could be contributing to the larger number of elderly patients being biopsied in the Northern Territory.

In this study, a slightly higher incidence of renal diseases was noted in female (male 44.6% female 55.4%). On the contrary, males outnumbered females in most study population as demonstrated in multiple studies and registry data globally [8, 9]. The female excess in kidney diseases, was not completely understood. It might be explained by lower nephron endowment in females (estimated 17% less nephrons).

Sub-nephrotic proteinuria was the most common (47.76%) clinical indication of renal biopsy in our study. This was followed by nephrotic syndrome (35.82%) and nephritic syndrome (30.45%). This trend was different to the other available studies worldwide including Jalalah at Saudi Arabia and Mardanour et al. at Iran [10, 11]. Nephritic syndrome contributed to 30.44% of the renal biopsy in our study. Acute kidney injury contributed to 28.06% of the renal biopsy in our study. However, AKI was present in only 1.8% patients in the study by Narsimham et al. and 0.9% in the Japanese registry data. Our result was similar to Pakistan (20.1%) and South Africa (21.3%). This wide variation could be explained by the varying incidence of AKI and the difference in approaching AKI locally [8].

We identified diabetes nephropathy as the leading pathological findings, followed by FSGS. The prevalence of DMN varies across geographies, ranging from 0.15–5%. DMN accounted for 1.6% of all glomerular diseases in an India study. In our trial, 59 indigenous and 12 non- indigenous cases were found to have diabetes nephropathy. 28.2% was indigenous males while 54.9% was indigenous females.

IgA nephropathy is the most common primary, biopsy-proven glomerulonephritis in six out of the eight national registries (Italy, Spain, Czech Republic, Denmark, Scotland, Japan) and regions like Victoria in Australia, Japan, China and Singapore, with total diagnoses ranging from 12.6 to 45%. In our study, IgA nephropathy contributed to 7.9% (*n* = 44), consisting of 15.9% indigenous males, 22.7% indigenous females, 38.6% non-indigenous males and 22.7% non-indigenous females.

In Brazil (24.6%) and Bahrain (23.8%), focal segmental glomerulonephritis was the most common primary GN. 47 cases in our study has FSGS, contributing to 8.5%. Indigenous females have the highest number (*n* = 15, 31.9%), followed by non-indigenous female (*n* = 13, 27.7%), indigenous male (*n* = 11, 23.4%) and non-indigenous male (*n* = 8, 17.0%). Our prevalence of FSGS is significantly higher than most centres of China, but lower than that East China from Hou’s study (7.3%) [9, 12–14].

This study showed several practical prognostic findings. We showed that indigenous population had significant less favourable overall survival outcome than non-indigenous population. Indigenous population had a higher dialysis rate than non-indigenous population. Besides, indigenous population who had at least one episode of dialysis had significant worse outcome than non-indigenous population. This survival disparities were well described in multiple papers on other comorbidities (i.e. cancer) by Condon et.al and Moore et.al [10, 11]. Factors like miscommunication and social cultural differences, poor understanding of medical advice and geographic isolation might be causing the treatment disparity [10].

The strength of this study is that this cohort study is a single centre research with relatively large sample size and long duration. It is the largest renal biopsy study in the region. We aim to help in the future research on the development of further diagnostic and therapeutic advancement. With analysis of indigeneity for each histopathology diagnosis, we can clearly visualize the difference in histopathological pattern between indigenous and non-indigenous.

This study is limited by its retrospective design and the fact that renal biopsy may not represent the complete information as to the aetiologies of patient’s renal disease. We did not audit biopsy results and independently confirm recorded diagnosis for this study. Another limitation is the difficult comparison with the other registries and centres because of the potential differences in biopsy rates with geographical variation. Besides, the reported incidences are usually based on moderate and severe diseases. Thus, mild forms of renal disease may be underdiagnosed. The analysis of histopathology was done in an ideal world situation where one biopsy has one diagnosis. In biopsies with more than one features/ diagnosis, a predominant feature would be selected based on the pathology report.

This database will help the Top End Health to define the phenotypic and histologic disease pattern for specific entities such as DMN and establish longitudinal patient cohorts for further research. It will also serve as a database for further studies and clinical programs to improve the early diagnosis and outcome of common diseases like IgA nephropathy, DMN and FSGS.

## Conclusion

To conclude, diabetic nephropathy is the most common native renal biopsy diagnosis in the Northern Territory, followed by focal segmental glomerulosclerosis and IgA nephropathy. Besides, there is higher proportional mortality among indigenous population than non-indigenous population in the Top-End. This study supports the speculation of survival disparities among indigenous and excess of diabetic nephropathy in the Northern Territory. In turn, this study warrants some diabetic patients (especially among indigenous population) who had atypical features to have a kidney biopsy promptly.

## Data Availability

The databases used and/or analysed during the current study are available from the corresponding author on reasonable request.

## References

[1] Luciano RL, Moeckel GW (2019). Update on the native kidney biopsy: core curriculum 2019. Am J Kidney Dis.

[2] Charles Darwin University. A multicultural northern territory- statistics from the 2016 census, Available at: https://www.cdu.edu.au/northern-institute/multicultural-northern-territory. (Accessed: 4 Dec 2020).

[3] Cunningham A, Benediktsson H, Muruve DA, Hildebrand AM, Pietro RP (2018). Trends in biopsy-based diagnosis of kidney disease: a population study. Can J Kidney Health Dis.

[4] Hu R, Quan S, Wang Y, Zhou Y (2020). Spectrum of biopsy proven renal diseases in Central China: a 10 year retrospective study based on 34,630 cases. Nat Res.

[5] Habib MA, Badruddoza SM (2012). Pattern of glomerular diseases among adults in Rajshahi, the Northern region of Bangladesh. Saudi J Kidney Dis Transpl.

[6] Islam SMJ, Haque WS, Akhter S, MahbubulAlam SM (2018). Histomorphological pattern of renal biopsy in dhaka: a single center study. Saudi J Kidney Dis Transpl.

[7] Mohammad N, Khan TM, Orakzai AN, Imran M (2012). Histological pattern of glomerulopathies. Gomal J Med Sci.

[8] Narasimhan B, Chacko B, John GT, Korula A, Kirubakaran MG, Jacob CK (2006). Characteristization of kidney lesions in Indian adults: towards a renal biopsy registry. J Nephrol.

[9] Tyagi I, Majumdar K, Kamra S, Batra VV (2013). Retrieval of kidney tissue for light microscopy from frozen tissue processed for immunofluorescence: a simple procedure to avoid repeat kidney biopsies. Indian J Nephrol.

[10] Moore SP, Green AC, Bray F. et al. Survival disparities in Australia: an analysis of patterns of care and comorbidities among indigenous and non-indigenous cancer patients. BMC Cancer. 14;517(2014). 10.1186/1471-2407-14-517 10.1186/1471-2407-14-517 PMC4223410 25037075

[11] Condon JR, Armstrong BK, Barnes T, Zhao Y (2005). Cancer incidence and survival for indigenous Australians in the Northern Territory. Aust N Z J Public Health..

[12] Mittal P, Agarwal SK, Singh G, Bhowmik D, Mahajan S, Dinda A, Bagchi S (2020). Spectrum of biopsy proven renal disease in northern India: a single centre study. Nephrology.

[13] Jalalah SM (2009). Patterns of primary glomerular diseases among adults in the western region of Saudi Arabia. Saudi J Kidney Dis Transpl.

[14] Mardanpour K, Rahbar M (2013). Histopathologic patterns of adult renal disease in Kermanshah, Iran: a 6-year review of two referral centers. Caspian J Intern Med.

